# Evaluation of oral health status in the population above 50: evidence from the ardakan cohort study on aging (ACSA)

**DOI:** 10.1186/s12903-024-03916-4

**Published:** 2024-01-31

**Authors:** Ahmad Delbari, Fatemeh Ghavidel, Vahid Rashedi, Mohammad Bidkhori, Mohammad Saatchi, Elham Hooshmand

**Affiliations:** 1 Iranian Research Center on Aging, University of Social Welfare and Rehabilitation Sciences, Evin, Daneshjoo Boulevard, Koodakyar Street, Tehran, Iran; 2 Iranian Research Center on Aging, Department of Aging, University of Social Welfare and Rehabilitation Sciences, Tehran, Iran; 3 Department of Biostatistics and Epidemiology, University of Social Welfare and Rehabilitation Science, Tehran, Iran; 4 Health in Emergency and Disaster Research Center, University of Social Welfare and Rehabilitation Sciences, Tehran, Iran

**Keywords:** DMF Index, Oral health, Epidemiology, Older adults

## Abstract

**Background:**

The global population is undergoing rapid aging, and older individuals are more susceptible to various health issues, including oral health problems. Despite the increasing attention given to healthy aging, oral health has often been overlooked in discussions related to health problems. This study aims to assess the oral health status of middle-aged and older adults in Iran.

**Methods:**

This cross-sectional study analyzed data from 4970 men and women aged 50 years and above, who participated in the Ardakan Cohort Study on Aging (ACSA) between 2020 and 2022. Trained personnel administered a questionnaire and conducted oral health examinations to determine the oral health status and oral hygiene behaviors of the participants.

**Results:**

The mean value (SD) of the total Decayed, Missing, and Filled Teeth (DMFT) index was 21.3 (10.7). Approximately 46% of all participants were completely edentulous (without natural teeth). Moreover, 58.5% of the total sample wore dentures, and the mean age (SD) when they started using dentures was 48.5 (20.7) years. About 71% of participants with natural teeth reported brushing their teeth at least once a day, while about 63% of denture wearers cleaned their dentures daily. In the sample, 28% of individuals had visited a dentist in the last year. The prevalence of difficulty in biting and chewing food among the participants was 48.2% and 44.6%, respectively. Additionally, nearly 68% of all samples reported experiencing at least one difficulty in daily functioning due to oral/dental conditions. The study identified cutoff points of seven (sensitivity = 56.8 and specificity = 77.5) and 10 (sensitivity = 72.1 and specificity = 71.1) missing teeth, indicating the presence of at least one problem in daily functioning due to oral/dental conditions in middle-aged and older adults, respectively.

**Conclusion:**

The study reveals a high prevalence of edentulism and denture use among participants aged 50 years and above. The majority of samples reported difficulties in daily functioning due to oral and dental conditions, especially in biting and chewing food. These findings highlight the importance of proactive measures to address oral health issues in middle-aged and older adults, thereby enhancing their overall health and well-being.

## Introduction

The world’s population is aging rapidly, with the number of older adults predicted to be from 1 billion to 2.1 billion between 2019 and 2050, according to the World Health Organization (WHO) [1]. Like other countries, Iran is facing population aging, which has important implications for the health and well-being of its older adults. Recent data shows that the proportion of Iranians aged 60 years and over is currently around 10%, expected to increase to 21.7% by 2050 [2].

These demographic shifts have serious implications for healthcare systems, as the older population is more susceptible to various health issues, including oral health problems. Tooth loss, tooth decay, periodontal disease, dry mouth, and oral cancer are among the most prevalent oral health problems experienced by older adults [3]. A systematic review study in 2022 showed that 1.1–70% of people aged 45 years and above were edentulism [4]. In a recent systematic review comprising 13 studies, the prevalence of edentulism among older adults in Iran was found to be 48% [5].

Poor oral health can lead to a range of complications, including difficulties with swallowing, chewing, and speech [6]. Additionally, poor oral health has been linked to sleep disturbances and reduced work productivity [7, 8]. Research has shown that edentulous individuals are at a higher risk of having poor diets and inadequate nutrition compared to those with natural teeth [9–11]. Tooth loss is associated with an increased risk of ischemic stroke and poor mental health [12]. Periodontal disease has been linked to a range of health problems, including diabetes and ischemic heart disease [13–15]. These relationships underscore the importance of maintaining good oral health as part of a comprehensive approach to overall health and well-being.

To comprehensively understand the prevalence and distribution of oral health problems in the general population of Iran, it is crucial to conduct more epidemiological studies using larger and representative sample sizes. This is particularly important because much of the existing research on oral health in Iran has been clinical and based on convenience samples. The objective of this study was to examine the oral health status and health behaviors of a representative sample of individuals aged 50 years and above residing in Ardakan, Iran.

## Method

### Study design and population

This cross-sectional study utilizes data from the first wave of the Ardakan Cohort Study on Aging (ACSA). The IRanian Longitudinal Study on Aging (IRLSA) is the framework for conducting the ACSA. More details regarding the study design can be found in previous publications [16]. Briefly, in 2020, the ACSA was launched to understand various aspects of aging in Ardakan, a region located in central Iran. This cohort comprises community-dwelling adults aged ≥ 50 years who were recruited through multistage stratified random sampling and applied a series of questionnaires as well as laboratory and clinical examinations. Regarding sampling, health centers were conceptualized as a stratum. To select a proportionate sample in each stratum a complete list of people over 50 years covered by each health center was taken. Subsequently, participants were randomly selected from health centers in each stratum. The inclusion criteria for ACSA were individuals who were 50 years or older and residing in Ardakan City. Exclusion criteria included a diagnosis of dementia, deafness, blindness, mental or psychological disorders such as mental retardation or psychosis, paralysis, and inability to understand and respond to study questions. All ACSA participants, whose oral examination data and questionnaire were available, were included in the present study.

### Oral health assessment

All dental examinations were conducted in-person at the cohort site by a trained dental hygienist who was calibrated to evaluate the indices. The DMFT index was used to assess Decayed, Missed, and Filled teeth, with the WHO’s criteria for caries diagnosis utilized. The criteria included identifying lesions in points, grooves, or smooth surfaces, temporary or permanent fillings, and holes in proximal surfaces to diagnose decayed teeth. Teeth that had been extracted were recorded as missing [17]. Besides that, the number of natural teeth was also counted. Based on this count, participants were grouped into one of four categories: those with no natural teeth, those with 1–9 natural teeth, those with 10–19 natural teeth, and those with 20 or more natural teeth.

To assess the participants’ subjective evaluation of the status of their teeth and gums, they were asked, “How do you generally evaluate the condition of your teeth/gums?“. The three items that are used to evaluate oral health-related behaviors are (1) Use of dental floss, (2) Use of mouthwash, and (3) Brushing habits. In addition, the study included a question specifically addressing the cleaning practices of participants who wear full dentures, asking about the methods they use to clean their dentures, such as brushing or other techniques. Dental visits and any dental or oral pain/discomfort experienced during the last 12 months were assessed through self-report. Participants self-reported their use of removable dentures and were categorized into one of five groups: (1) Partial denture, (2) Full upper denture, (3) Full lower denture, (4) Both upper and lower dentures, or (5) No dentures worn. Also, the age of starting to wear the denture was asked.

A specific item was used to assess the Oral Impact on Daily Performance (OIDP). The OIDP is a global socio-dental indicator assessing the impact of oral health conditions on individuals’ daily activities and quality of life [18]. This questionnaire was adapted in Iran in 2007 [19]. In the present study, OIDP items were selected by a dentist and two gerontologists. According to the aim of the study to propose a cut point for missing teeth that may be related to the daily functioning of middle-aged and older adults, especially their nutrition and social activity, OIDP items were selected. The responses to this item are scored on a scale of 0 to 4, with response options ranging from never (0) to very often [4].

### Baseline characteristics

Participants were interviewed with standardized questionnaires to find out baseline characteristics by staff trained. To examine socioeconomic status, this study considered indicators such as age, sex, marital status, and economic status. The economic status was measured by asking respondents, “In general, how do you evaluate your economic situation?“.

Smoking status was evaluated into three categories as “never smoker”, “former smoker” or “current smoker”. Body max index (BMI) was calculated using the following formula: weight (kg)/height squared (m2). The participants were classified into four categories according to BMI: underweight (BMI ≤ 18 kg/m2); normal (18.5 kg/m2 ≤ BMI < 25 kg/m²); overweight (BMI ≥ 25 and < 30 kg/m²); and obesity (BMI ≥ 30 kg/m²).

### Statistical analysis

Descriptive statistics were used to present sample characteristics, including means and standard deviations (SD) for continuous variables, and percentages or frequency for categorical variables. Quantitative data were analyzed using either a t-test or an Analysis of Variance (ANOVA), depending on the number of groups being compared. Categorical variables were analyzed using the chi-square test to assess participant differences. To assess the missing teeth number could identify people who experience a problem due to oral condition, sensitivity, and specificity were calculated. Discrimination was assessed by the Receiver Operating Characteristic (ROC) curve. The corresponding sensitivity (Sen) and specificity (Spe) for the specific cutoff point were then determined. The interpretation of the Area Under the ROC Curve (AUC) could be stated as not discriminative if 0.5 to 0.7, acceptable 0.7 to 0.8, excellent if 0.8 to 0.9, and outstanding if more than 0.9 [20]. The optimal cutoff score for the assessment tool was determined by Youden’s J statistic. STATA Statistical Software Version 15 (Stata-Corp. 2017) and R software (R-4.3.1) were used to perform all data analyses and statistical significance was set to α = 0.05.

## Results

As of the current investigation, data from 5197 ACSA participants were accessible. Following the exclusion of missing cases, the final analysis involved 4970 (95.6%) individuals. Out of 4,970 participants in this study, 2,581 (51.9%) were females, and 4,522 (91%) were married. About 64.5% (*n* = 3,206) were aged between 50 and 64 years, and 35.5% (*n* = 1,764) were 65 and above years. It was observed that 75.5% (*n* = 3,744) of the participants were never-smokers, 10.8% (*n* = 535) were categorized as former smokers, and only 13.6% (*n* = 675) of them were current smokers. In addition, more than two-thirds of the participants are in the overweight and obese group (*n* = 3,347). The mean age of starting to use dentures (partial or complete) in this sample was 48.55 (20.7) years (Table 1).

**Table 1:** Baseline Characteristics of participants above 50 in the ardakan cohort study on aging (ACSA) (*n* = 4,970)

Variable	Level	N (%) or mean (SD)
**Age**	50–64	3,206 (64.5%)
	65≤	1,764 (35.5%)
**Sex**	Female	2,581 (51.9%)
	Male	2,389 (48.1%)
**Marital status**	Single	448 (9.0%)
	Couple	4,522 (91.0%)
**Educational level**	No formal education	702 (14.1%)
	Elementary	2,378 (47.9%)
	Middle	724 (14.6%)
	High School	594 (12.0%)
	University	569 (11.4%)
**Economic status**	Highest	22 (0.4%)
	High	345 (7.0%)
	Middle	2,432 (49.3%)
	Low	1,223 (24.8%)
	Lowest	911 (18.5%)
**BMI**	Underweight	47 (1.1%)
	Normal	998 (22.5%)
	Overweight	1,847 (41.7%)
	Obese	1,540 (34.7%)
**Age of starting to use dentures**	-	48.55 (± 20.7)

SD: Standard Deviation, BMI: Body Max Index

The mean values of the decayed teeth, missing teeth, and filled teeth indices among the subjects were 5.4 (6.1), 11.9 (9.1), and 4.0 (5.2), respectively. Additionally, the mean DMFT index was 21.3 (10.7), with females having a higher mean (22.2 ± 11.0) than males (20.1 ± 10.4), and this difference was statistically significant (*P* < 0.001). Out of the sample, nearly half 2,289 (46%) of the participants were edentulism. More than half of the male subjects 1,076 (50.7%) and 1,213 (41.7%) of the female subjects were edentulism. In addition, the prevalence of edentulous was 33.9% (*n* = 1,202) for adults aged 50–64 years, and about 68.1% (*n* = 1,202) for adults ≥ 65 years (*p*-value < 0.001). This study found that approximately 45% (*n* = 2,247) of all participants wear removable dentures in the full upper and lower. It was observed that about 38% (*n* = 1,897) of all samples reported experiencing dental/oral pain or discomfort in the previous 12 month. The results revealed significant differences between sex groups, with females (*n* = 1,156, 44%) reporting a higher frequency of dental/oral pain or discomfort compared to males (*n* = 741, 31%) (*p*-value < 0.001). About 48% (*n* = 2,380) reported good dental health, and 60% (*n* = 2,991) reported good gum health (Table 2).

**Table 2:** Oral/dental health status of participants above 50 in the ardakan cohort study on aging (ACSA) (*n* = 4,970)

Variables	Total	Age			Sex		
		50–64	65≤	***p***-value	Female	Male	***p***-value
**DMFT Index**	21.3 (10.7)	20.95 (10.96)	22.61 (9.88)	**< 0.001**	22.2 (11.0)	20.1 (10.4)	**< 0.001**
Decayed	5.4 (6.1)	5.73 (6.12)	4.39 (5.60)		5.7 (6.1)	5.1 (5.8)	
Missing	11.9 (9.1)	10.70 (8.38)	16.12 (10.11)		11.8 (8.6)	11.9 (9.5)	
Filled	4.0 (5.2)	4.52 (5.40)	2.09 (3.69)		4.6 (5.7)	3.1 (4.2)	
**Number of natural teeth**							
No natural teeth	2,289 (46.1%)	1,087 (33.9%)	1,202 (68.1%)	**< 0.001**	1,076 (41.7%)	1,213 (50.7%)	**< 0.001**
1–9	425 (8.5%)	272 (8.5%)	153 (8.7%)		232 (8.6%)	202 (8.5%)	
10–19	662 (13.3%)	481 (15.0%)	181 (10.3%)		392 (15.2%)	270 (11.3%)	
20 or more	1,594 (32.1%)	1,366 (42.6%)	228 (12.9%)		890 (34.5%)	704 (29.5%)	
**Presence of removable dentures**							
Partial removable denture	483 (9.7%)	384 (12.0%)	99 (5.6%)	**< 0.001**	288 (11.2%)	195 (8.1%)	**< 0.001**
Full upper denture	138 (2.8%)	102 (3.2%)	36 (2.0%)		96 (3.7%)	42 (1.8%)	
Full lower denture	35 (0.7%)	23 (0.7%)	12 (0.1%)		27 (1.0%)	8 (0.3%)	
Both upper and lower dentures	2,247 (45.2%)	1,088 (33.9%)	1,159 (65.7%)		1,061 (41.1%)	1,186 (49.7%)	
No dentures worn	2,067 (41.6%)	1,609 (50.2%)	458 (26.0%)		1,109 (43.0%)	958 (40.1%)	
**Status of dental health**							
Good	2,380 (47.9%)	1,458 (45.5%)	922 (52.3%)	**< 0.001**	1,112 (43.1%)	1,268 (53.1%)	**< 0.001**
Fair	1,721 (34.6%)	1,186 (37.0%)	535 (30.3%)		992 (38.4%)	729 (30.5%)	
Poor	869 (17.5%)	562 (17.5%)	307 (17.4%)		477 (18.5%)	392 (16.4%)	
**Status of gum health**							
Good	2,991 (60.1%)	1,976 (61.6%)	1,015 (57.5%)	**< 0.001**	1,423 (55.2%)	1,559 (65.3%)	**< 0.001**
Fair	1,181 (23.8)	774 (14.1%)	407 (23.1%)		654 (25.5%)	527 (22.1%)	
Poor	798 (16.1%)	456 (14.2%)	342 (19.4%)		495 (19.3%)	303 (12.6%)	
**Mouth sores**	247 (5.0%)	154 (4.8%)	93 (5.3%)	0.467	166 (6.4%)	81 (3.4%)	**< 0.001**
**Dental visit during the last 12 months (yes)**	1,391 (28%)	1,099 (34.3%)	292 (16.5%)	**< 0.001**	783 (38.2%)	511 (25.1%)	**< 0.001**
**Dental/oral pain or discomfort during the last 12 months (yes)**	1,897 (38.2%)	1,361(42.4%)	536 (30.4%)	**< 0.001**	1,156 (44.8%)	741 (31.0%)	**< 0.001**

The dental hygiene habits of the study sample are presented in Table 3. Of the participants with natural teeth, 23.1% (*n* = 630) reported using floss to clean their teeth, while only 2.6% (*n* = 71) reported using mouthwash. Most of them reported using a toothbrush once or multiple times a day (*n* = 2,075, 75.5%). Additionally, 62.7% (*n* = 1,408) of people without natural teeth reported using a toothbrush or other methods for cleaning their teeth.

**Table 3:** Frequency and percentage of dental hygiene habits (tooth cleaning) of participants above 50 in the ardakan cohort study on aging (ACSA) (*n* = 4,970)

Variables	Total	Age			Sex			
		50–64	65≤	***p***-value		Female	Male	***p***-value
**With natural teeth**								
Flossing (yes)	631 (23.5%)	538 (25.4%)	93 (16.5%)	**< 0.001**		397 (26.4%)	234 (19.9%)	**< 0.001**
Mouthwash (yes)	71 (2.6%)	61 (2.9%)	10 (1.6%)	0.875		44 (2.9%)	27 (2.3%)	0.315
Brushing Habits (yes)	1,910 (71.2%)	1,551 (73.2%)	359 (63.9%)	**0.026**		1,152 (76.5%)	758 (64.4%)	**< 0.001**
**Without natural teeth**								
Cleaning dentures (yes)	1,374 (62.1%)	681 (64.2%)	693 (60.2%)	**< 0.001**		717 (68.7%)	657 (56.1%)	**< 0.001**

Table 4 presents an overview of the Oral health problems experienced during the previous 12 months in the different ages and sex groups. Regarding the oral health problem, biting difficulty was reported as the most common problem across all ages and genders categorized (*n* = 2,397, 48.2%). Chewing difficulty was the second most common item of OIDP (*n* = 2,219, 44.6%). Speech/word pronouncing difficulties (*n* = 716, 14.4%), feeling embarrassed (*n* = 767, 15.4%), avoiding smiling (*n* = 740, 14.1%), and a decrease in social activities (*n* = 188, 3.8%) were reported less than others.

**Table 4:** Frequency and percentage of oral health problems experienced during the previous 12 month in participants above 50 in the ardakan cohort study on aging (ACSA) (*n* = 4,970)

Variables	Total	Age			Sex		
		50–64	65≤	***p***-value	Female	Male	***p***-value
**Biting difficulty**	2,397 (48.2%)	1,378 (43.0%%)	1,019 (57.8%)	**< 0.001**	1,352 (52.4%)	1,045 (43.8%)	**< 0.001**
**Difficulty chewing food**	2,219 (44.6%)	1,399 (43.6%)	820 (46.5%)	0.265	1,296 (50.2%)	923 (38.6%)	**< 0.001**
**Speech/word pronouncing difficulties**	716 (14.4%)	430 (13.4%)	286 (16.2%)	**0.002**	383 (14.8%)	333 (14%)	0.366
**Felt embarrassed due to appearance of teeth**	767 (15.4%)	584 (18.2%)	183 (10.4%)	**< 0.001**	480 (18.6%)	287 (12%)	**< 0.001**
**Avoid smiling**	740 (14.1%)	531 (16.6%)	173 (9.8%)	**< 0.001**	463 (17.9%)	241 (10.1%)	**< 0.001**
**Decrease in social activities due to oral and dental conditions**	188 (3.8%)	149 (4.6%)	39 (2.2%)	**< 0.001**	95 (3.7%)	93 (3.9%)	0.695
**Having** **at least one problem OIDP**	3,174 (63.8%)	1,980 (61.8%)	1,194 (67.7%)	**< 0.001**	1,774 (68.7%)	1,400 (58.6%)	**< 0.001**

OIDP: Oral Impact on Daily Performance

**Fig. 1:**
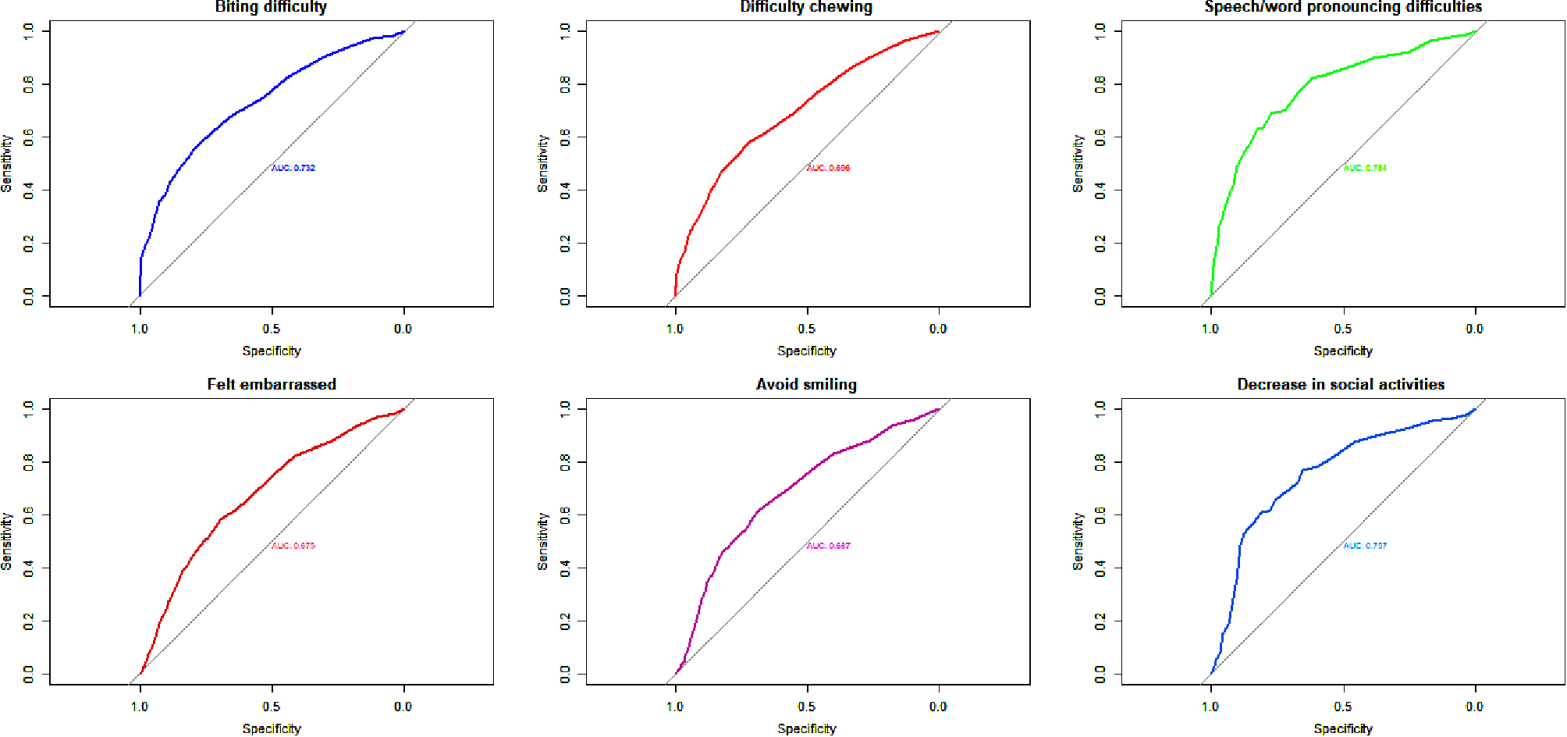
Receiver-operator characteristic (ROC) curves for the missing teeth for OIDP items in without denture 50–64 in the ardakan cohort study on aging (ACSA) (*n* = 1,609)

**Fig. 2:**
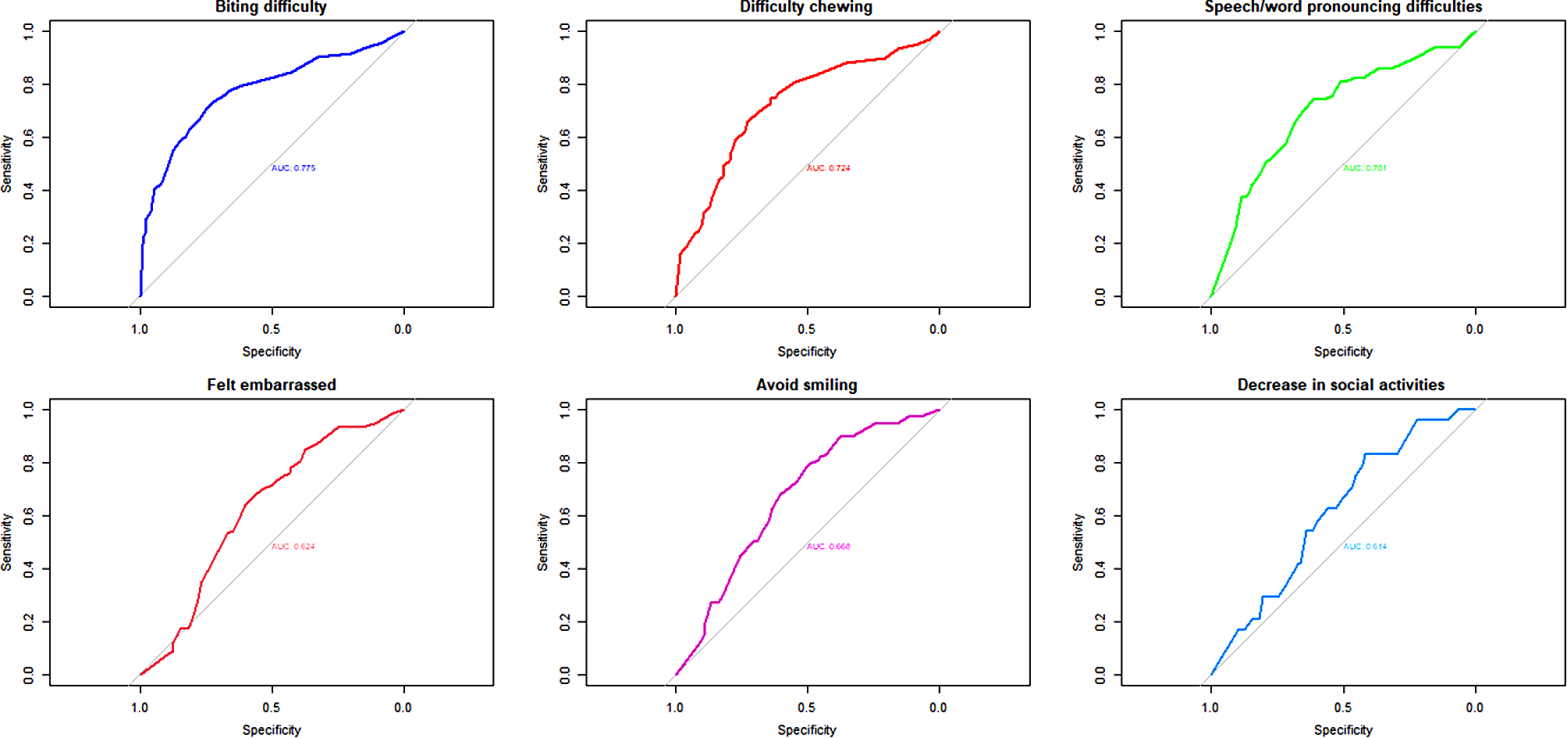
Receiver-operator characteristic (ROC) curves for the missing teeth for OIDP items in without denture participants aged ≥ 65 in the ardakan cohort study on aging (ACSA) (*n* = 485)

Table 5 provides the recommended thresholds for the number of missing teeth by oral/dental problems in subjects without dentures. The cut-off value of missing teeth for difficulty biting food was reported to be 10 for people aged 50–64 years (Sen = 55.2 and Spe = 79.9) and 15 for people 65 years and above (Sen = 70.6 and Spe = 74.9). The cut-off values for the decrease in social activities due to oral/dental conditions in middle-aged and older adults were eight (Sen = 77.0 and Spe = 65.4) and 10 (Sen = 83.3 and Spe = 41.9), respectively. Finally, the cut-off points for having at least one problem because of oral and dental conditions was reported to be seven in the middle-aged (Sen = 56.8 and Spe = 77.5), while it was 10 for the older adults (Sen = 72.1 and Spe = 71.1). In additional, ROC curves were constructed separately for middle-aged (Fig. 1) and older adults (Fig. 2) using items of OIDP.

**Table 5:** The characteristics of different cut points for missing teeth vs. oral impact on daily performance

	50–64				65≤				
OIDP items		Cut-point	Sensitivity (CI 95%)	Specificity (CI 95%)	The area under ROC curve (CI 95%)	Cut-point	Sensitivity (CI 95%)	Specificity (CI 95%)	The area under ROC curve (CI 95%)
**Biting difficulty**		> 10	55.2 (50.8 to 59.6)	79.9 (77.4 to 82.2)	0.723 (0.710 to 0.754)	> 15	70.6 (64.2 to 76.4)	74.9 (68.7 to 80.4)	0.775 (0.733 to 0.812)
**Difficulty chewing food**		> 9	50.8 (47.2 to 54.4)	79.1 (76.2 to 81.8)	0.696 (0.673 to 0.719)	> 14	65.8 (59.9 to 71.4)	72.8 (65.7 to 79.1)	0.724 (0.681 to 0.765)
**Speech/word pronouncing difficulties**		> 12	68.9 (60.4 to 76.6)	77.3 (75.0 to 79.4)	0.784 (0.763 to 0.804)	> 16	74.4 (63.2 to 83.6)	61.2 (56.1 to 66.1)	0.701 (0.657 to 0.743)
**Felt embarrassed due to the appearance of teeth**		> 8	58.1 (52.9 to 63.3)	69.3 (66.7 to 71.9)	0.675 (0.651 to 0.698)	> 16	64.1 (53.5 to 73.9)	60.1 (54.9 to 65.2)	0.624 (0.577 to 0.668)
**Avoid smiling**		> 8	81.6 (55.8 to 67.3)	68.5 (66.0 to 71.0)	0.687 (0.663 to 0.709)	> 13	78.2 (67.4 to 86.8)	50.6 (45.5 to 55.8)	0.668 (0.623 to 0.711)
**Decrease in social activities due to oral and dental conditions**		> 8	77.0 (66.8 to 85.4)	65.4 (63.0 to 67.8)	0.757 (0.735 to 0.778)	> 10	83.3 (62.6 to 95.3)	41.9 (37.2 to 46.7)	0.614 (0.568 to 0.659)
**Having at least one oral impact on daily performance**		> 7	56.8 (53.5 to 60.0)	77.5(74.2 to 80.6)	0.725 (0.703 to 0.747)	> 10	72.1 (66.9 to 77.0)	71.1 (62.7 to 78.6)	0.758 (0.716 to 0.796)

OIDP: Oral Impact on Daily Performance, IC: Confidence Interval, ROC: Receiver-operator characteristic

## Discussion

This cross-sectional study aimed to determine the oral health status of community-dwelling adults aged ≥ 50 years living in Ardakan, Iran. The results demonstrated that the mean DMFT index was found to be 21.3. Nearly 46% of the participants were found to be edentulism. Moreover, a substantial proportion of the participants reported experiencing one or more oral health issues within the 12 months preceding the study.

Based on the findings of this study, the mean number of DMFT was calculated to be 21.3 (10.7). The missing component of the DMFT index was found to be the highest (11.9) for both sexes. In a similar investigation carried out in Iran by Hadilou et al. (2022), the mean DMFT value was documented as 21.27 (8.95) [21]. According to the study, the average number of missing teeth was higher than the average number of decayed and filled teeth. Also, several studies showed an extremely high MT score in DMFT components [22–24]. This may be attributed to the fact that as age, the number of remaining teeth tends to decrease, resulting in a lower incidence of dental caries and a reduced need for dental restorations such as fillings.

In the current study, the prevalence of edentulism was 46%, with an age-related increase. Specifically, individuals aged 50–64 reported about 40% prevalence, while those aged 65 and older showed a higher prevalence of 68%. A study conducted in South Korea revealed that 9.5% of the geriatric population were without teeth, and this percentage increased with increasing age, with 18.1% of participants aged 80 years being edentulism [25]. In a United States-based study, the prevalence of edentulism within the 65–74 age group was reported to be 13% [26]. According to a similar study conducted in Iran, the prevalence of edentulous individuals in the 50–60 age group was reported to be 21% [7]. The last Iranian national oral health survey conducted and published by the Ministry of Health and Medical Education in 2012, indicated that the prevalence of edentulism exceeded 50% in the 65–74 age group [27]. The high prevalence of edentulous among the study participants may be due to several factors. Tooth extraction is often chosen due to its affordability compared to more expensive dental procedures like root canals or implants, aligning with limited budgets. Notably, health insurance in Iran covers only a fraction of dental costs. The preference for extraction may also stem from a fear of complex procedures, lack of awareness about alternative treatments, or insufficient understanding of long-term consequences like jawbone loss. Additionally, in Iranian culture, dentures are widely accepted among older adults, contributing to the prevalent choice of extraction and dentures over other dental interventions [28–30].

Among the study sample, just 28% reported having had a dental visit in the past year. In the American study, 57% of the participants reported having had a dental visit within the past year [31]. The high prevalence of edentulism and the prevalence of using dentures among study subjects may contribute factors to the low proportion of dental visits reported in the study.

The study’s findings showed that about three-quarters of participants with natural teeth reported using a toothbrush at least once daily, while only 23.5% included flossing in their regular oral hygiene routine. According to a previous study on Iranian individuals aged 60–70 years, the authors reported that about 50% of the participants brushed their teeth once a day and only 20% brushed their teeth twice or more daily [32]. Among denture users, the study observed that roughly 63% reported employing a toothbrush or alternative cleaning methods to maintain denture hygiene at least once a day. The findings of this study emphasize the importance of teaching the proper way to clean teeth and dentures in middle-aged and older adults.

The problem of chewing and biting food was the most common problem among the participants. In a similar study conducted on the Iranian population, it was found that nearly half (47%) of individuals aged 50–65 years reported problems with biting, while more than half (54%) reported difficulty chewing food [33]. Research showed that tooth loss and the use of dentures may reduce chewing ability, and limit dietary choices [34]. Studies suggest that chewing with removable dentures maybe 30–40% less efficient than chewing with natural teeth [35]. According to a study conducted in multiple European countries, 10% of the participants, including denture wearers, reported reduced quality of life due to oral and dental problems [36]. Another study found that an increase in the number of lost or decayed teeth was associated with greater difficulty in eating fruits among the participants [37]. The study also found that decreased social activities were less commonly reported among subjects. This may be to the fact that a majority of participants used dentures, which may have improved their confidence in the appearance of their teeth and reduced any potential social stigma associated with missing or damaged teeth.

According to the study, it appears that having at least 22 and 23 natural teeth in the middle-aged may be associated with better performance in biting and chewing food properly, respectively. While older adults, having at least 17 natural teeth for biting and 18 natural teeth for chewing food without problems may be necessary. WHO suggested that having 20 teeth is considered necessary for functional dentition [38]. However, the ability to bite and chew food depends on various factors, including the location and distribution of teeth, the condition of the remaining teeth, and other oral and systemic health conditions [39–41]. The observed difference in the cut-off point for the number of missing teeth between these two age groups may be at least partially influenced by the impact of tooth loss on food choices. Tooth loss can make it difficult to bite and chew certain types of food, which may lead to a preference for easier-to-chew foods that are high in saturated fats and lacking in important nutrients such as carotenes, vitamin C, and fiber [11, 42].

According to a study conducted by Astrøm et al., OIDP increased rapidly for Norwegian subjects with less than 28 natural teeth [43]. The study findings suggest that there is a difference in the cut-off point for the number of missing teeth that shows at least one problem in daily functioning between middle-aged and older individuals. Specifically, the cut-off point was reported as seven for middle-aged individuals and 10 for older adults. However, it is important to note that these differences may be because the problems were self-reported, and individuals may have different standards for their health status depending on their age. In other words, older individuals may attribute their loss of ability in the reported fields to their age rather than to oral conditions. Therefore, it is important to consider these factors when interpreting the study’s findings. The cause of tooth loss may differ between middle-aged and older adults, with factors such as periodontal disease, dental caries, and trauma being more common in middle-aged individuals, while tooth loss because of aging and systemic diseases may be more prevalent among older adults [44]. It’s also important to note that the cut-off points reported in the study are based on statistical analysis and should be interpreted with caution.

### Limitations and strengths

This study makes a valuable contribution to the existing literature on the impact of natural teeth and dentures on oral health and daily performance in middle-aged and older adults. The study’s strengths include its large sample size, consideration of a different aspects of oral status. The findings of the study can provide important insights for researchers and policymakers in developing effective interventions and policies to improve oral health outcomes for older adults. Nevertheless, it had some limitations. First, some of the data collected was based on self-reported information, which may have introduced bias into the results. Second, the cut point for problems related to oral conditions did not consider edentulism participants, while wearing dentures can also cause issues such as difficulty in biting and chewing.

## Conclusion

In conclusion, this study sheds light on the considerable prevalence of edentulous individuals and denture usage among the participants, particularly among those aged 60 years and above. Additionally, the findings emphasize the noteworthy impact of oral health on everyday functioning, as many subjects reported challenges with biting and chewing food. These results underscore the critical need for proactive measures to address oral health concerns, aiming to enhance overall health and well-being among middle-aged and older adults.

## Data Availability

The datasets analyzed during the current study are available from the corresponding author on reasonable request.

## References

[1] World Health Organization. Ageing. 2022. Available from: https://www.who.int/news-room/fact-sheets/detail/ageing-and-health. Accessed Jun 2023.

[2] Goharinezhad S, Maleki M, Baradaran HR, Ravaghi H. A qualitative study of the current situation of elderly care in Iran: what can we do for the future? Glob Health Action. 2016;9:32156. 27876456 10.3402/gha.v9.32156 PMC5120385

[3] Murray Thomson W. Epidemiology of oral health conditions in older people. Gerodontology. 2014;31(s1):9–16. 24446974 10.1111/ger.12085

[4] Borg-Bartolo R, Roccuzzo A, Molinero-Mourelle P, Schimmel M, Gambetta-Tessini K, Chaurasia A, et al. Global prevalence of edentulism and dental caries in middle-aged and elderly persons: a systematic review and meta-analysis. J Dent. 2022;127:104335. 36265526 10.1016/j.jdent.2022.104335

[5] Rabiei M, Masoudi Rad H, Homaie Rad E, Ashourizadeh S. Dental status of the Iranian elderly: a systematic review and meta-analysis. J Invest Clin Dent. 2019;10(4):e12459. 10.1111/jicd.12459 31628734

[6] Rosenoer L, Sheiham A. Dental impacts on daily life and stisfaction with teeth in relation to dental status in adults. J Rehabil. 1995;22(7):469–80. 10.1111/j.1365-2842.1995.tb01191.x 7562211

[7] Jahangiry L, Bagheri R, Darabi F, Sarbakhsh P, Sistani MMN, Ponnet K. Oral health status and associated lifestyle behaviors in a sample of Iranian adults: an exploratory household survey. BMC Oral Health. 2020;20(1):82. 32192497 10.1186/s12903-020-01072-z PMC7082917

[8] Aida J, Kondo K, Yamamoto T, Hirai H, Nakade M, Osaka K, et al. Oral health and cancer, cardiovascular, and respiratory mortality of Japanese. J Dent Res. 2011;90(9):1129–35. 21730255 10.1177/0022034511414423

[9] Moynihan PJ, Snow S, Jepson NJ, Butler TJ. Intake of non-starch polysaccharide (dietary fibre) in edentulous and dentate persons: an observational study. Br Dent J. 1994;177(7):243–7. 7917631 10.1038/sj.bdj.4808575

[10] Nowjack-Raymer RE, Sheiham A. Association of edentulism and diet and nutrition in US adults. J Dent Res. 2003;82(2):123–6. 12562885 10.1177/154405910308200209

[11] Walls AW, Steele JG, Sheiham A, Marcenes W, Moynihan PJ. Oral health and nutrition in older people. J Public Health Dent. 2000;60(4):304–7. 11243051 10.1111/j.1752-7325.2000.tb03339.x

[12] Joshipura KJ, Hung H-C, Rimm EB, Willett WC, Ascherio A. Periodontal Disease, tooth loss, and incidence of ischemic stroke. Stroke. 2003;34(1):47–52. 12511749 10.1161/01.str.0000052974.79428.0c

[13] Grossi SG, Genco RJ. Periodontal disease and diabetes mellitus: a two-way relationship. Ann Periodontol. 1998;3(1):51–61. 9722690 10.1902/annals.1998.3.1.51

[14] Shlossman M, Knowler WC, Pettitt DJ, Genco RJ. Type 2 diabetes mellitus and periodontal disease. J Am Dent Assoc. 1990;121(4):532–6. 2212346 10.14219/jada.archive.1990.0211

[15] Joshipura KJ, Rimm EB, Douglass CW, Trichopoulos D, Ascherio A, Willett WC. Poor oral health and coronary heart disease. J Dent Res. 1996;75(9):1631–6. 8952614 10.1177/00220345960750090301

[16] Aminisani N, Azimi-Nezhad M, Shamshirgaran SM, Mirhafez SR, Borji A, Poustchi H, et al. Cohort Profile: the IRanian longitudinal study on Ageing (IRLSA): the first comprehensive study on ageing in Iran. Int J Epidemiol. 2022;51(4):e177–e88. 35137100 10.1093/ije/dyab272

[17] World Health Organization. Oral health surveys: basic methods. 2013. Available from: https://www.who.int/publications/i/item/9789241548649. Accessed Jun 2023.

[18] Adulyanon S, Vourapukjaru J, Sheiham A. Oral impacts affecting daily performance in a low dental disease Thai population. Community Dent Oral Epidemiol. 1996;24(6):385–9. 9007354 10.1111/j.1600-0528.1996.tb00884.x

[19] Dorri M, Sheiham A, Tsakos G. Validation of a persian version of the OIDP index. BMC Oral Health. 2007;7:2. 17257407 10.1186/1472-6831-7-2 PMC1797167

[20] Hosmer DW Jr, Lemeshow S, Sturdivant RX. Applied logistic regression: John Wiley & Sons; 2013.

[21] Hadilou M, Somi MH, Faramarzi E, Nikniaz L. Effect of Beverage Consumption frequency on DMFT Index among Iranian Adult Population: an AZAR Cohort Study. Int J Dent. 2022;2022:9142651. 35669588 10.1155/2022/9142651 PMC9167004

[22] Chikte U, Pontes CC, Karangwa I, Kimmie-Dhansay F, Erasmus R, Kengne AP, Matsha TE. Dental caries in a South African adult population: findings from the Cape Town Vascular and Metabolic Health Study. Int Dent J. 2020;70(3):176–82. 31808148 10.1111/idj.12538 PMC9379168

[23] Msyamboza KP, Phale E, Namalika JM, Mwase Y, Samonte GC, Kajirime D, et al. Magnitude of dental caries, missing and filled teeth in Malawi: national oral Health Survey. BMC Oral Health. 2016;16(1):1–6. 26956884 10.1186/s12903-016-0190-3 PMC4784360

[24] Tanık A. Evaluation of the relationship of CPITN and DMFT index of adult patients in Turkey with their demographic characteristics: an epidemiological study. Biotechnol Biotechnol Equip. 2019;33(1):1626–34.

[25] Park H-A, Shin S-H, Ryu J-I. Edentulous disparities among geriatric population according to the sexual difference in South Korea: a nationwide population-based study. Sci Rep. 2023;13(1):7854. 37188776 10.1038/s41598-023-35029-3 PMC10185582

[26] Dye B, Thornton-Evans G, Li X, Iafolla T. Dental caries and tooth loss in adults in the United States, 2011–2012. NCHS Data Brief. 2015(197):197. 25973996

[27] Khoshnevisan MH, Ghasemianpour M, Samadzadeh H, Baez RJ. Oral Health Status and Healthcare System in I.R. Iran. J Contemp Med Sci. 2018;4(3).

[28] Kalsbeek H, Truin GJ, Burgersdijk R, van’t Hof M. Tooth loss and dental caries in Dutch adults. Commun Dent Oral Epidemiol. 1991;19(4):201–4. 10.1111/j.1600-0528.1991.tb00146.x 1889191

[29] Soh G, Chong Y, Ong G. Dental state and needs for episodic care of institutionalized elderly in an Asian community. Soc Sci Med. 1992;34(4):415–8. 1566122 10.1016/0277-9536(92)90301-6

[30] Kamberi B, Koçani F, Begzati A, Kelmendi J, Ilijazi D, Berisha N, Kqiku L. Prevalence of Dental Caries in Kosovar Adult Population. Int J Dent. 2016;2016:4290291. 27516774 10.1155/2016/4290291 PMC4969505

[31] Burgette JM, Lee JY, Baker AD, Vann WF. Jr. Is Dental Utilization Associated with oral health literacy? J Dent Res. 2016;95(2):160–6. 26567035 10.1177/0022034515617457 PMC4720957

[32] Asgari F, Majidi A, Koohpayehzadeh J, Etemad K, Rafei A. Oral hygiene status in a general population of Iran, 2011: a key lifestyle marker in relation to common risk factors of non-communicable diseases. Int J Health Policy Manag. 2015;4(6):343–52. 26029893 10.15171/ijhpm.2015.18 PMC4450729

[33] Jahangiry L, Bagheri R, Darabi F, Sarbakhsh P, Sistani MMN, Ponnet K. Oral health status and associated lifestyle behaviors in a sample of Iranian adults: an exploratory household survey. BMC Oral Health. 2020;20:1–9. 10.1186/s12903-020-01072-z PMC7082917 32192497

[34] Åstrøm A, Haugejorden O, Skaret E, Trovik T, Klock K. Oral impacts on daily performance in Norwegian adults: the influence of age, number of missing teeth, and socio-demographic factors. Eur J Oral Sci. 2006;114(2):115–21. 16630302 10.1111/j.1600-0722.2006.00336.x

[35] Idowu A, Handelman S, Graser G. Effect of denture stability, retention, and tooth form on masticatory function in the elderly. Gerodontics. 1987;3(4):161–4. 3326781

[36] Eurobarometer S. 330. Oral Health. 2010.

[37] Mehrstedt M, John MT, Tönnies S, Micheelis W. Oral health-related quality of life in patients with dental anxiety. Community Dent Oral Epidemiol. 2007;35(5):357–63. 17822484 10.1111/j.1600-0528.2007.00376.x

[38] Organization WH. Recent advances in oral health: report of a WHO expert committee [meeting held in Geneva from 3 to 9 December. 1991]: World Health Organization; 1992. 1462607

[39] Figueiredo Dde R, Peres MA, Luchi CA, Peres KG. [Chewing impairment and associated factors among adults]. Rev Saude Publica. 2013;47(6):1028–38. 24626541 10.1590/S0034-8910.2013047004789 PMC4206107

[40] Sun-Waterhouse D, Kang W, Ma C, Waterhouse GIN. Towards human well-being through proper chewing and safe swallowing: multidisciplinary empowerment of food design. J Future Foods. 2021;1(1):1–24.

[41] Shiozawa K, Ohnuki Y, Mototani Y, Umeki D, Ito A, Saeki Y, et al. Effects of food diameter on bite size per mouthful and chewing behavior. J Physiological Sci. 2016;66(1):93–8. 10.1007/s12576-015-0411-6 PMC10717241 26493202

[42] Ervin RB, Dye BA. The effect of functional dentition on healthy eating Index scores and nutrient intakes in a nationally representative sample of older adults. J Public Health Dent. 2009;69(4):207–16. 19453869 10.1111/j.1752-7325.2009.00124.x

[43] Åstrøm A, Haugejorden O, Skaret E, Trovik T, Klock K. Oral impacts on daily performance in Norwegian adults: validity, reliability and prevalence estimates. Eur J Oral Sci. 2005;113(4):289–96. 16048520 10.1111/j.1600-0722.2005.00225.x

[44] Gabiec K, Bagińska J, Łaguna W, Rodakowska E, Kamińska I, Stachurska Z et al. Factors Associated with tooth loss in General Population of Bialystok, Poland. Int J Environ Res Public Health. 2022;19(4). 10.3390/ijerph19042369 PMC8872086 35206557

