# Correction: Evaluation of oral health status in the population above 50: evidence from the ardakan cohort study on aging (ACSA)

**DOI:** 10.1186/s12903-024-05023-w

**Published:** 2024-10-15

**Authors:** Ahmad Delbari, Fatemeh Ghavidel, Vahid Rashedi, Mohammad Bidkhori, Mohammad Saatchi, Elham Hooshmand

**Affiliations:** 1 Iranian Research Center on Aging, University of Social Welfare and Rehabilitation Sciences, Tehran, Iran; 2 Iranian Research Center on Aging, Department of Aging, University of Social Welfare and Rehabilitation Sciences, Tehran, Iran; 3 Department of Biostatistics and Epidemiology, University of Social Welfare and Rehabilitation Science, Tehran, Iran; 4 Health in Emergency and Disaster Research Center, University of Social Welfare and Rehabilitation Sciences, Tehran, Iran

**Correction to: BMC Oral Health (2024) 24:154**

10.1186/s12903-024-03916-4.

Following the original publication [1], the author would like to correct the incorrect ethics code and the mistake in affiliations.

Correct Ethics code: IR.USWR.REC.1399.490

The correct affiliations 1 and 2 are given below:

1) Iranian Research Center on Aging, University of Social Welfare and Rehabilitation Sciences, Tehran, Iran

2) Iranian Research Center on Aging, Department of Aging, University of Social Welfare and Rehabilitation Sciences, Tehran, Iran
